# Genome Sequence of a Sulfate-Reducing Thermophilic Bacterium, Thermodesulfobacterium commune DSM 2178^T^ (Phylum *Thermodesulfobacteria*)

**DOI:** 10.1128/genomeA.01490-14

**Published:** 2015-01-29

**Authors:** Srijak Bhatnagar, Jonathan H. Badger, Ramana Madupu, Hoda M. Khouri, Elizabeth M. O’Connor, Frank T. Robb, Naomi L. Ward, Jonathan A. Eisen

**Affiliations:** a Microbiology Graduate Group, University of California Davis, Davis, California, USA; b J. Craig Venter Institute, La Jolla, California, USA; c J. Craig Venter Institute, Rockville, Maryland, USA; d Institute of Marine and Environmental Technology, and Department of Microbiology and Immunology, University of Maryland, Baltimore, Maryland, USA; e Department of Molecular Biology, University of Wyoming, Laramie, Wyoming, USA; f UC Davis Genome Center, Department of Evolution and Ecology and Department of Medical Microbiology and Immunology, University of California Davis, Davis, California, USA

## Abstract

Here, we present the complete genome sequence of Thermodesulfobacterium commune DSM 2178^T^ of the phylum *Thermodesulfobacteria*.

## GENOME ANNOUNCEMENT

Thermodesulfobacterium commune is the type species of genus Thermodesulfobacterium contained within the small class *Thermodesulfobacteria*, and first isolated from Ink Pot spring in Yellowstone National Park, WY, in 1980. It is a sulfate-reducing obligate anaerobic thermophile with an optimum growth temperature of 70°C and a growth temperature range of 45°C to 80°C. This Gram-negative bacterium has nonmotile, non-cyst-forming, nonsporulating, straight, rod-shaped (0.3 × 0.9 µm) cells (1). It can metabolize lactate and pyruvate as energy sources using sulfate and thiosulfate as electron acceptors (1). T. commune contains cytochrome c3 but lacks desulfoviridin-type bisulfate reductase (2). It was sequenced as part of the “Assembling the Tree of Life” project at The Institute of Genomic Research (TIGR). It was chosen as a representative of the phylum *Thermodesulfobacteria*, which had no sequenced members during the starting phase of the project (2002).

The type strain of T. commune (DSM 2178^T^) was obtained from the Leibniz Institute DSMZ-German Collection of Microorganisms and Cell Cultures and grown under 95% N_2_/5% CO_2_ atmosphere at 70°C using DSMZ medium 206. DNA was obtained by solubilizing cells with N-lauryl sulfate and sodium dodecyl sulfate followed by incubation with proteinase K. The lysate was extracted with Tris-EDTA-saturated phenol, chloroform/isoamyl alcohol, and was precipitated from the aqueous phase with 95% ethanol. It was resolubilized, incubated with DNase-free RNase and further purified by cesium-chloride gradient centrifugation and visualized using 365-nm UV light (3). Pulse-field gel electrophoresis was used to confirm the size and uniformity of the DNA preparation. Genome sequencing was performed as for the other genomes from the Tree of Life project (4). It included insert libraries of three different sizes: small (2 to 3 kb), medium (4 to 5 kb), and large (8 to 10 kb), which were sequenced with Sanger sequencing and assembled as previously described (5–7); assemblies were edited and gaps were closed by clone walking and targeted PCR and sequencing. Finishing was completed by (i) generating additional coverage in low coverage regions, (ii) verification of repeats, and (iii) resolution of ambiguities (8). The final assembly had ~9× coverage for the 1,764,045-bp genome with a GC content of 36.97%.

The origin of replication was identified using GC skew and colocalization of origin-associated genes (9). All the universal single-copy bacterial marker genes (10) were found in the sequenced genome using Phylosift (11). The genome was annotated using NCBI Prokaryotic Genome Annotation Pipeline version 2.6 (revision 438450) (12, 13). Of the 1,532 genes identified, 1,453 were protein-coding sequences (CDS), 29 were pseudogenes, and 50 were noncoding RNA genes. The 50 RNA genes comprise 1 noncoding RNA (ncRNA), 3 rRNAs (5S, 16S, and 23S), and 46 tRNAs. Additionally, two clustered regularly interspaced short palindromic repeat (CRISPR) arrays were identified in the genome.

### Nucleotide sequence accession number.

The genome sequence has been deposited at GenBank under the accession no. CP008796.

## References

[1] Zeikus JG, Dawson MA, Thompson TE, Ingvorsen K, Hatchikian EC 1983 Microbial ecology of volcanic sulphidogenesis: isolation and characterization of *Thermodesulfobacterium commune* gen. nov. and sp. nov. Microbiology 129:1159–1169. doi:10.1099/00221287-129-4-1159.

[2] Hatchikian EC, Zeikus JG 1983 Characterization of a new type of dissimilatory sulfite reductase present in *Thermodesulfobacterium commune*. J Bacteriol 153:1211–1220. 6826522 10.1128/jb.153.3.1211-1220.1983 PMC221765

[3] Charbonnier F, Forterre P 1995 Purification of plasmids from thermophilic and hyperthermophilic archaea, p 87–90. In Robb FT, Place AR (ed), Archaea: a laboratory manual Thermophiles. Cold Spring Harbor Laboratory, Cold Spring Harbor, NY.

[4] Wu D, Raymond J, Wu M, Chatterji S, Ren Q, Graham JE, Bryant DA, Robb F, Colman A, Tallon LJ, Badger JH, Madupu R, Ward NL, Eisen JA 2009 Complete genome sequence of the aerobic CO-oxidizing thermophile *Thermomicrobium roseum*. PLoS One 4:e4207. doi:10.1371/journal.pone.0004207. 19148287 PMC2615216

[5] Wu D, Daugherty SC, Van Aken SE, Pai GH, Watkins KL, Khouri H, Tallon LJ, Zaborsky JM, Dunbar HE, Tran PL, Moran NA, Eisen JA 2006 Metabolic complementarity and genomics of the dual bacterial symbiosis of sharpshooters. PLoS Biol 4:e188. doi:10.1371/journal.pbio.0040188. 16729848 PMC1472245

[6] Heidelberg JF, Paulsen IT, Nelson KE, Gaidos EJ, Nelson WC, Read TD, Eisen JA, Seshadri R, Ward N, Methe B, Clayton RA, Meyer T, Tsapin A, Scott J, Beanan M, Brinkac L, Daugherty S, DeBoy RT, Dodson RJ, Durkin AS, Haft DH, Kolonay JF, Madupu R, Peterson JD, Umayam LA, White O, Wolf AM, Vamathevan J, Weidman J, Impraim M, Lee K, Berry K, Lee C, Mueller J, Khouri H, Gill J, Utterback TR, McDonald LA, Feldblyum TV, Smith HO, Venter JC, Nealson KH, Fraser CM 2002 Genome sequence of the dissimilatory metal ion-reducing bacterium *Shewanella oneidensis*. Nat Biotechnol 20:1118–1123. doi:10.1038/nbt749. 12368813

[7] Heidelberg JF, Seshadri R, Haveman SA, Hemme CL, Paulsen IT, Kolonay JF, Eisen JA, Ward N, Methe B, Brinkac LM, Daugherty SC, Deboy RT, Dodson RJ, Durkin AS, Madupu R, Nelson WC, Sullivan SA, Fouts D, Haft DH, Selengut J, Peterson JD, Davidsen TM, Zafar N, Zhou L, Radune D, Dimitrov G, Hance M, Tran K, Khouri H, Gill J, Utterback TR, Feldblyum TV, Wall JD, Voordouw G, Fraser CM 2004 The genome sequence of the anaerobic, sulfate-reducing bacterium *Desulfovibrio vulgaris* Hildenborough. Nat Biotechnol 22:554–559. doi:10.1038/nbt959. 15077118

[8] Tettelin H, Radune D, Kasif S, Khouri H, Salzberg SL 1999 Optimized multiplex PCR: efficiently closing a whole-genome shotgun sequencing project. Genomics 62:500–507. doi:10.1006/geno.1999.6048. 10644449

[9] Lobry JR 1996 Asymmetric substitution patterns in the two DNA strands of bacteria. Mol Biol Evol 13:660–665. doi:10.1093/oxfordjournals.molbev.a025626. 8676740

[10] Wu D, Jospin G, Eisen JA 2013 Systematic identification of gene families for use as “markers” for phylogenetic and phylogeny-driven ecological studies of bacteria and archaea and their major subgroups. PLoS One 8:e77033. doi:10.1371/journal.pone.0077033. 24146954 PMC3798382

[11] Darling AE, Jospin G, Lowe E, Matsen FA, Bik HM, Eisen JA 2014 PhyloSift: phylogenetic analysis of genomes and metagenomes. PeerJ 2:e243. doi:10.7717/peerj.243. 24482762 PMC3897386

[12] Angiuoli SV, Gussman A, Klimke W, Cochrane G, Field D, Garrity G, Kodira CD, Kyrpides N, Madupu R, Markowitz V, Tatusova T, Thomson N, White O 2008 Toward an online repository of standard operating procedures (SOPs) for (meta)genomic annotation. Omics J Integr Biol 12:137–141. doi:10.1089/omi.2008.0017. PMC3196215 18416670

[13] Tatusova T, DiCuccio M, Badretdin A, Chetvernin V, Ciufo S, Li W 2013 Prokaryotic genome annotation pipeline. In Beck J, Benson D, Coleman J, Hoeppner M, Johnson M, Maglott M, Mizrachi I, Morris R, Ostell J, Pruitt K, Rubinstein W, Sayers E, Sirotkin K, Tatusova T (ed), The NCBI handbook, 2nd ed., National Center for Biotechnology Information, Bethesda, MD.

